# Assessment of Cancer-Related Fatigue, Pain, and Quality of Life in Cancer Patients at Palliative Care Team Referral: A Multicenter Observational Study (JORTC PAL-09)

**DOI:** 10.1371/journal.pone.0134022

**Published:** 2015-08-05

**Authors:** Satoru Iwase, Takashi Kawaguchi, Akihiro Tokoro, Kimito Yamada, Yoshiaki Kanai, Yoshinobu Matsuda, Yuko Kashiwaya, Kae Okuma, Shuji Inada, Keisuke Ariyoshi, Tempei Miyaji, Kanako Azuma, Hiroto Ishiki, Sakae Unezaki, Takuhiro Yamaguchi

**Affiliations:** 1 Department of Palliative Medicine, Institute of Medical Science, The University of Tokyo, Tokyo, Japan; 2 Department of Practical Pharmacy, Tokyo University of Pharmacy and Life Sciences, Tokyo, Japan; 3 Department of Psychosomatic Internal Medicine, National Hospital Organization Kinki-Chuo Chest Medical Center, Sakai, Japan; 4 Department of Breast Surgery, Tokyo Medical University Hospital, Tokyo, Japan; 5 Department of Palliative Medicine, The University of Tokyo Hospital, Tokyo, Japan; 6 Department of Palliative Medicine, Tokyo Medical University Hospital, Tokyo, Japan; 7 Department of Radiology, The University of Tokyo Hospital, Tokyo, Japan; 8 Department of Psychosomatic Medicine, The University of Tokyo Hospital, Tokyo, Japan; 9 Japanese Organisation for Research and Treatment of Cancer (JORTC), NPO, Tokyo, Japan; 10 Department of Clinical Trial Data Management, Graduate School of Medicine, The University of Tokyo, Tokyo, Japan; 11 Department of Pharmacy, Tokyo Medical University Hospital, Tokyo, Japan; 12 Division of Biostatistics, Tohoku University Graduate School of Medicine, Sendai, Japan; Postgraduate Institute of Medical Education and Research, INDIA

## Abstract

**Introduction:**

Cancer-related fatigue greatly influences quality of life in cancer patients; however, no specific treatments have been established for cancer-related fatigue, and at present, no medication has been approved in Japan. Systematic research using patient-reported outcome to examine symptoms, particularly fatigue, has not been conducted in palliative care settings in Japan. The objective was to evaluate fatigue, pain, and quality of life in cancer patients at the point of intervention by palliative care teams.

**Materials and Methods:**

Patients who were referred to palliative care teams at three institutions and met the inclusion criteria were invited to complete the Brief Fatigue Inventory, Brief Pain Inventory, and European Organisation for Research and Treatment of Cancer Quality of Life Questionnaire-Core 15-Palliative.

**Results:**

Of 183 patients recruited, the majority (85.8%) were diagnosed with recurrence or metastasis. The largest group (42.6%) comprised lung cancer patients, of whom 67.2% had an Eastern Cooperative Oncology Group Performance Status of 0–1. The mean value for global health status/quality of life was 41.4, and the highest mean European Organisation for Research and Treatment of Cancer Quality of Life Questionnaire-Core 15-Palliative symptom item score was for pain (51.0). The mean global fatigue score was 4.1, and 9.8%, 30.6%, 38.7%, and 20.8% of patients’ fatigue severity was classified as none (score 0), mild (1–3), moderate (4–6), and severe (7–10), respectively.

**Discussion:**

Cancer-related fatigue, considered to occur more frequently in cancer patients, was successfully assessed using patient-reported outcomes with the Brief Fatigue Inventory for the first time in Japan. Results suggested that fatigue is potentially as problematic as pain, which is the main reason for palliative care.

## Introduction

Cancer-related fatigue (CRF) is defined as “a physical, emotional, and/or perceived fatigue or tiredness, with a persistent and subjective sense related to cancer or cancer treatments that is disproportionate to recent activities and interferes with usual functioning.”[1] The prevalence of fatigue among advanced cancer patients or patients with bone metastasis is reported to be at least 75%, indicating a strong influence upon their quality of life (QOL) [2]. The frequency rate for the occurrence bone pain has been found to be approximately 40% in breast cancer patients with bone metastasis [3]. The proportion of patients complaining of bone pain and fatigue is high in those with metastatic cancer. However, there have been no studies conducted to assess the relationship between pain severity and fatigue using comprehensive assessment tools. There are many unknown elements regarding fatigue among terminally ill cancer patients. CRF can be categorized as either primary or secondary [4]. Primary fatigue is related to the tumor itself or cytokine derived from cancer treatment. Secondary fatigue is regarded as a disease-related symptom such as sleep disturbance, infection, malnutrition, or anemia. Although CRF greatly influences QOL in cancer patients, no specific treatments have been established for this type of fatigue, and currently, no medication has been approved in Japan.

There is evidence that 4 mg dexamethasone is effective for cancer-related fatigue [5]. Therefore, dexamethasone is expected to be available for the treatment of fatigue. However, more than 4 mg dexamethasone is usually used in chemotherapy to prevent allergy. There is also evidence of the effectiveness of psychostimulant agents for cancer-related fatigue [6], but frequent use is not recommended. The present consensus is that a safe and effective medication for CRF has not been found.

In Japan, Morita et al. researched various symptoms experienced by patients, which were estimated by a palliative care team (PCT) and a palliative care unit, using the Japanese version of the Support Team Assessment Schedule (STAS), in which symptom levels are rated using a scale ranging from 0 to 4 [7]. The prevalences of pain, fatigue, appetite loss, insomnia, and somnolence experienced by patients and assessed by the PCT were 100%, 50%, 93%, 80%, and 82%, respectively. After 7 days of PCT intervention, there were significant improvements in pain, appetite loss, and insomnia scores relative to those at initial assessment. In contrast, fatigue and somnolence, for which treatment has not been established, did not improve significantly. However, the STAS used by Morita el al. [7] is a proxy assessment tool, and systematic research using patient-reported outcome (PRO) to examine symptoms, particularly fatigue, has not been conducted in Japan. In addition, there may be a large gap between PRO and clinician-reported outcome (CRO) in measures such as the STAS. PRO has been used in previous fatigue studies, but there is no gold standard.

Minton et al. recommend the use of the Fundamental Assessment of Cancer Therapy: Fatigue questionnaire and the European Organisation for Research and Treatment of Cancer Quality of Life Questionnaire-Core 30 (EORTC QLQ-C30) to assess CRF [8]. In addition, the National Comprehensive Cancer Network (NCCN) reviewed the Brief Fatigue Inventory (BFI) and Fundamental Assessment of Cancer Therapy: Fatigue questionnaire, which resulted in development of a new scale known as the NCCN Fatigue Contributing Factors Inventory [9]. The BFI, presently one of the most commonly used scales, is a convenient questionnaire with which to assess CRF. This is also used to determine the severity of fatigue occurring during the preceding 24 hours and the interference it causes in the individual’s life.

This study investigated relationships between fatigue, pain, and QOL by focusing on fatigue in PROs. We selected the BFI, Brief Pain Inventory (BPI), and European Organisation for Research and Treatment of Cancer Quality of Life Questionnaire-Core 15-Palliative (EORTC QLQ-C15-PAL) as assessment tools and included the Pain Management Index (PMI) to evaluate the correlation between inadequate analgesia and QOL. Our expectation was that fatigue assessed via an appropriate tool would be as prevalent as pain and exert an impact on QOL. The objective of the study was to evaluate patients’ QOL and the prevalence of fatigue and pain at the point of PCT intervention, in order to elucidate relationships between fatigue, pain, and QOL.

## Materials and Methods

### Ethical Considerations

The protocol for this observational study was prepared according to the Helsinki Declaration and the Ethical Guidelines for Epidemiology Research [10]. Ethical approval was granted by the IRB of National Hospital Organization Kinki-Chuo Chest Medical Center, Tokyo Medical University Hospital and The University of Tokyo Hospital. All patients were informed of the nature and purpose of the study, in writing and orally, and provided informed consent to participate in the study.

### Study Participants

The following inclusion criteria were applied: 1) aged between 20 and 80 years, 2) a performance status (PS) of 0–3 on the Eastern Cooperative Oncology Group (ECOG) scale, 3) a diagnosis of any type of cancer, and 4) ability to complete the questionnaire unaided. The exclusion criteria were as follows: 1) presence of dementia, agitation, or delirium; 2) presence of a mental disorder; and 3) an investigator’s decision that a request for survey participation would be inappropriate (e.g., due to a problematic, though not severe, psychiatric concern).

### Questionnaire Survey

Of the patients referred to PCTs at three institutions between May 2012 and August 2013, 185 (mean age = 63.5 years, SD = 11.2, age range: 35–88 years; 112 [61.2%] men) met the inclusion criteria and were invited to complete self-rated questionnaires to determine QOL and fatigue and pain levels.

### Outcome Measures

#### Brief Fatigue Inventory (BFI)

The BFI is a questionnaire, originally developed in English, for which validity and reliability have been verified. It is designed to assess fatigue in cancer patients [11]. Okuyama et al. confirmed the validity of the Japanese version of the BFI [12]. It consists of 9 items, which are rated using Likert scales ranging from 0 to 10. Average scores for these 9 items are used as global fatigue scores (GFSs); scores of 1–3, 4–6, and 7–10 are categorized as mild, moderate, and severe, respectively.

### European Organisation for Research and Treatment of Cancer Quality of Life Questionnaire-Core 15-Palliative

The EORTC QLQ-C15-PAL was developed by reducing the EORTC QLQ-C30, which is a comprehensive evaluation tool for cancer patients receiving palliative care [13]. The EORTC QLQ-C30 is used worldwide to assess QOL in cancer patients [14]. The validity of the Japanese version of the EORTC QLQ-C15-PAL was confirmed by Miyazaki et al. [15] The EORTC QLQ-C15-PAL consists of 15 items including a global health status/QOL item, a 5-item functioning subscale (assessing physical, role, emotional, cognitive, and social functioning), and a 9-item symptom subscale (assessing fatigue, nausea and vomiting, pain, dyspnea, insomnia, appetite loss, constipation, diarrhea, and financial difficulties). The functioning and symptom subscale item scores range from 0 to 100. In the functioning subscale, higher scores (≥60) indicate better functioning, whereas lower scores (≤40) indicate better physical condition in the symptom subscale.

#### Brief Pain Inventory (BPI)

The BPI is a comprehensive assessment tool for pain, developed by the Pain Research Group of the World Health Organization Collaborating Centre for Symptom Evaluation in Cancer Care [16]. The validity of the Japanese version was confirmed by Uki et al. [17] The BPI is used to measure pain severity and its interference with QOL and consists of 15 items concerning presence (1 item), sites (1 item) and severity of pain (4 items), status (1 item) and effects of pain treatment (1 item), and interference of pain in QOL (7 items). In this study, item 3 (severity of the worst pain experienced in the previous 24 hours) was only used to consider the burden that pain placed upon patients; this approach is recommended by the US Food and Drug Administration for using the tool in clinical studies.

#### Pain Management Index (PMI)

The PMI is a measure of the appropriateness of pharmacological strategies and compares the potency of prescribed analgesic medications using patient-reported pain levels. Analgesics were classified into four levels: 0: no analgesic; 1: nonopioid; 2: weak opioid; 3: strong opioid. Pain levels were scored as follows: 1–3: mild; 4–7: moderate; and 8–10: severe. PMI scores are calculated by subtracting patient-reported pain levels from analgesic levels, which produces a score range of -3 to 3. Negative scores (<0) indicate inadequate analgesia, and positive scores (≥0) indicate adequate analgesia [18].

#### Statistical analysis

The mean scores for each of the 9 BFI items were calculated to produce GFSs, for which mean values were obtained. The proportions of patients in the mild, moderate, and severe fatigue groups were also calculated. Summary statistics were produced for QOL (EORTC PAL-15) and pain (BPI and PMI) upon PCT referral, and the relationship between these scores and fatigue was discussed. Spearman’s correlation coefficients were estimated to determine associations between measured items. JMP-Pro 11 (SAS Institute Inc.) was used in the analysis.

## Results

### Study Participants

Of the 185 registered patients included in the study, data from two, who did not complete the BFI questionnaire, were excluded. Table 1 summarizes the patients’ characteristics. The majority of the patients (85.8%) were diagnosed with recurrence or metastasis. The largest group (43.2%) comprised lung cancer patients, 67.2% of whom had an ECOG PS of 0–1. Approximately 45% of the patients had taken anticancer medication within the previous month, and 42.1% were receiving chemotherapy. The median value of time since diagnosis (metastasis diagnosis to assessment date), was 4 months, and the quartile points were 0 and 11.5 months (interquartile range: 0–152 months).

**Table 1:** Patient Characteristics.

N = 183	Mean	SD	Range
Age	63.5	11.2	35–88
	N (%)		
Sex			
Male	112 (61.2)		
Female	71 (38.8)		
Type of cancer			
Lung	79 (43.2)		
Head and neck	17 (9.3)		
Pancreas	22 (12.0)		
Other	65 (35.5)		
Tumor burden			
Distant metastasis	142 (77.6)		
Local recurrence	15 (8.2)		
No	26 (14.2)		
Bone metastasis			
Yes	65 (35.5)		
No	116 (63.4)		
Unknown	2 (1.1)		
ECOG performance status			
0	22 (12.0)		
1	101 (55.2)		
2	37 (20.2)		
3	23 (12.6)		
Anticancer treatment within a month			
Chemotherapy	75(41.0)		
Chemotherapy, radiotherapy	10 (5.5)		
Radiotherapy	10 (5.5)		
Surgery, radiotherapy	1 (0.5)		
Surgery	3 (1.6)		
No	84 (45.9)		
Currently receiving chemotherapy			
Yes	77 (42.1)		
No	106 (57.9)		

### QQL (EORTC PAL-15) and Global Fatigue Scores

Table 2 contains the results for EORTC PAL-15 items, item 3 of the BPI (BPI-3), the BFI, and GFS. The mean value and standard deviation (SD) for global health status/QOL was 41.4 (24.6). The highest mean score for EORTC QLQ-C15-PAL symptom items was for pain (51.0), followed by fatigue (50.6), appetite loss (43.5), insomnia (41.2), constipation (32.4), dyspnea (28.5), and nausea and vomiting (11.5).

**Table 2:** QOL (EORTC PAL-15) and Global Fatigue Scores.

n = 173–183	Mean	SD
GHS/QOL	41.4	24.6
Physical functioning	57.2	26.1
Emotional functioning	64.5	27.6
Fatigue	50.6	28.5
Nausea and vomiting	11.5	22.2
Pain	51.0	31.1
Dyspnea	28.5	31.7
Insomnia	41.2	32.3
Appetite loss	43.5	35.0
Constipation	32.4	32.4
BPI-3	4.9	3.0
BFI-1_now	3.8	2.5
BFI-2_average	3.8	2.4
BFI-3_worst	4.3	2.8
BFI-4_ general activity	4.0	2.9
BFI-5_mood	4.1	3.0
BFI-6_walking ability	4.1	3.2
BFI-7_normal work	4.8	3.5
BFI-8_relationships with other people	3.6	3.3
BFI-9_enjoyment of life	4.7	3.4
Global fatigue score	4.1	2.5

The mean (SD) GFS value was 4.1 (2.5), and the mean score (SD) for the worst fatigue experienced (BFI-3) was 4.3 (2.8). Regarding the influence of pain on daily life, the lowest and highest mean scores were found for “relationships with other people” (3.6) and “normal work” (4.8), respectively.

### Severity of Global Fatigue and Worst Pain (BPI-3)

Table 3 displays fatigue severity levels classified according to the GFS and BPI-3.

**Table 3:** Severity of Fatigue.

	Global Fatigue Score (n = 173)	BPI-3 (n = 181)
	n (%)	n (%)
None (0)	17 (9.8)	19 (10.5)
Mild (1−3)	53 (30.6)	46 (25.4)
Moderate (4−6)	67 (38.7)	50 (27.6)
Severe (7−10)	36 (20.8)	66 (36.5)

Moderate to severe fatigue and pain were reported by 59.5% and 64.1% of patients, respectively.

### Pain Management Index

The mean BPI-3 (the worst pain) score was 4.9 (SD 3.0). Table 4 shows the PMI results. The mean PMI score was -0.1 (SD 1.3), and approximately 40% of patients exhibited PMI scores of 0; that is, they were receiving inadequate analgesia. Strong opioids were administered to 62.7% of patients receiving adequate analgesia.

**Table 4:** Pain Management Index.

PMI	Mean: -0.1		SD: 1.3
	≥0 (Adequate) 110 (61.1%)		
	<0 (Inadequate) 70 (38.9%)		
	Adequate pain management	Inadequate pain management	
	(n = 110)	(n = 70)	
	n (%)	n (%)	
No analgesic (0)	11 (10.0)	28 (40.0)	
Nonopioid (I)	16 (14.5)	31 (44.3)	
Weak opioid (II)	14 (12.7)	11 (15.7)	
Strong opioid (III)	69 (62.7)	0 (0.0)	

### Correlations between Measured Items

The supporting information file (S1) summarizes the correlations between all measured items. GFS was strongly positively correlated with all BFI items (0.80–0.89). BFI-6 (walking ability) and nausea and vomiting (0.10) were weakly positively correlated, as were BFI-5 (mood) and dyspnea (0.15)

## Discussion

This study evaluated fatigue, pain, and QOL in cancer patients referred to PCTs in three acute care hospitals. Approximately 60% of the patients demonstrated worse than moderate fatigue (according to GFSs), indicating that such patients experience moderate to severe fatigue.

Although the proportion of patients experiencing CRF has been reported within a wide range (14–95%), to our knowledge, no Japanese studies have used PROs to assess global fatigue [19–22]. Our study used the BFI, which had been used worldwide at the time the intervention was implemented. The validity of the Cancer Fatigue Scale, an alternative tool for measuring fatigue and available in Japanese, has been verified [23]. However, we chose the BFI because it allows assessment of fatigue based on multidimensional aspects.

GFS results showed that moderate or severe CRF, was found in a high proportion of patients (59.5%). This indicated that the majority of patients referred to PCTs at acute care hospitals experience moderate to severe fatigue.

The BFI subscales, BFI-4 to BFI-9, and BFI-8 (relationships with other people) displayed the lowest score (mean: 3.6). This finding may have resulted from only observing inpatients. The hospital environment is presumed to limit relationships with others, which may have been reflected in the scores.

In our study, moderate to severe fatigue and pain were reported by approximately 60% and 64% of patients (Table 3). This indicates that palliative care physicians and nurses should presuppose the existence of high-grade fatigue and pain in PCT interventions for cancer patients.

A previous study, reported by PCTs and conducted at one of the hospitals involved in the current study (University of Tokyo Hospital), used a proxy the Support Team Assessment Schedule (STAS) [24]. Moderate or severe pain (score 2, limiting some activity) and CRF at the point of PCT intervention were 81.1% and 49.3%, respectively [25]. Morita et al. [7] reported that mean scores obtained via the STAS at the same point in their study were 2.3 for pain and 0.80 for CRF, which indicated that CRF was experienced slightly or occasionally with normal activity and no distress, as the score was less than 1. Basch et al. [26] reported a gap in assessment between patient- and clinician-reported adverse symptoms, using the Common Terminology Criteria for Adverse Events established by the National Cancer Institute. A larger gap was observed in assessment of severe (Grade 3) fatigue relative to that reported for nausea and pain. The STAS, the previous study involving CRO, and our results indicate that CRF is likely to have been underestimated by medical staff.

Lee et al. [27] reported that the domains included in the EORTC QLQ-C15-PAL, including fatigue, are possible prognostic factors. Assessment using a PRO tool verified for validity, such as the BFI, may be of great significance in measuring CRF, which showed a large gap between PROs and CROs.

In this study, PMI results (Table 4) showed that inadequate pain management was observed in 38.9% patients at the point of PCT intervention in the acute care hospitals. In contrast, 61.9% patients received adequate analgesia. Strong opioids were administered to 62.7% of these patients. These results suggest that, to a certain extent, pain treatment using opioids is successful in general acute care hospital wards. Patients are primarily referred to PCTs for pain management; the results of BPI-3 and the PMI reflect this.

With respect to symptom scores, average pain and fatigue scores were high at 51.0 and 50.6 respectively, and the emotional functioning score was the highest at 64.5. Pain and fatigue were strongly positively correlated, with a correlation coefficient of 0.4, and emotional functioning and pain and fatigue were strongly negatively correlated, with correlation coefficients of -0.4 and -0.5 respectively. In previous studies, pain, fatigue, and depression have been reported to form symptom clusters [28–31]. In our study, approximately 64% of cancer patients in an acute care hospital demonstrated moderate to severe pain, and a similar proportion complained of concurrent moderate or severe fatigue (Table 3). A similar tendency may have been found in this study. However, the emotional score in this study refers to emotional functioning, and depression was not assessed using this item. Therefore, “emotional” score results should be interpreted carefully.

Proportions of patients experiencing moderate and severe fatigue in GFSs and pain on the BPI (classified according to the PMI) were high at 59.5% and 64.1%, respectively. This suggests that fatigue is as problematic as pain for cancer patients hospitalized in acute care hospitals. As approximately 20% of patients experience severe fatigue, it is considered an important symptom that should not be ignored in PCT interventions.

Bone metastasis was observed in 35.5% of participants and is commonly assumed to be a cause of pain. All participating study sites were acute care hospitals, and a high proportion of patients, 42.1%, were treated with chemotherapy upon admission to the hospital. Therefore, PCTs in acute care hospitals have the greatest likelihood of observing adverse events. Regarding the timing of PCT intervention, the high proportion of patients with metastasis, at 77.6%, and the median value of 4 months since diagnosis indicated that early stage interventions had not yet been effective.

A radical treatment for fatigue is to eliminate the causes. However, in most cancer patients, causes can be specified but cannot be eliminated; therefore, drug treatment is chosen. Medications, such as psychostimulants, phytotherapeutic agents, growth factors, and corticosteroids, show evidence of positive effects [32], but no medication has currently been approved in Japan. This study suggests that CRF is a potential problem for cancer patients. Therefore, the development of medication for CRF is urgently required in Japan.

Finally, the data should be interpreted cautiously, with consideration of the possible omission of patients with poor PS. Further, patients without pain may not be referred to a PCT. Anemia, considered to exert a strong influence on fatigue, was not assessed in terminally ill cancer patients to avoid unnecessary blood collection. In this study, patients referred to the team were all registered; therefore, the influence of primary doctors’ intentions on the results is undeniable.

In conclusion, CRF, considered to occur more frequently in cancer patients, was successfully assessed using PROs with the BFI for the first time in Japan. When patients are referred to PCTs, many complain of fatigue. This suggests that fatigue is potentially as problematic as pain, which is the main reason for palliative care. As treatment choices for CRF are extremely limited in Japan, the research and development of treatments to reduce fatigue is urgently required.

## Supporting Information

S1 Database (XLSX)

S1 Dataset (XLSX)

S1 Table (DOCX)
