# Supplementary material for: Assessment of Cancer-Related Fatigue, Pain, and Quality of Life in Cancer Patients at Palliative Care Team Referral: A Multicenter Observational Study (JORTC PAL-09)
Source: PLoS One. 2015 Aug 5;10(8):e0134022. doi: 10.1371/journal.pone.0134022 (PMC4526465; doi:10.1371/journal.pone.0134022)
Supplement: S1 Table — (DOCX) [file pone.0134022.s003.docx]

Table 5. Correlations Between Measured Items

|  | GHS/QOL | Physical functioning | Emotional functioning | Fatigue | Nausea and vomiting | Pain | Dyspnea | Insomnia | Appetite loss | Constipation | BPI-3 | BFI-1 | BFI-2 | BFI-3 | BFI-4 | BFI-5 | BFI-6 | BFI-7 | BFI-8 | BFI-9 | Global Fatigue Score |
| --- | --- | --- | --- | --- | --- | --- | --- | --- | --- | --- | --- | --- | --- | --- | --- | --- | --- | --- | --- | --- | --- |
| GHS/QOL | 1 |  |  |  |  |  |  |  |  |  |  |  |  |  |  |  |  |  |  |  |  |
| Physical functioning | 0.49 | 1 |  |  |  |  |  |  |  |  |  |  |  |  |  |  |  |  |  |  |  |
| Emotional functioning | 0.44 | 0.37 | 1 |  |  |  |  |  |  |  |  |  |  |  |  |  |  |  |  |  |  |
| Fatigue | -0.5 | -0.54 | -0.52 | 1 |  |  |  |  |  |  |  |  |  |  |  |  |  |  |  |  |  |
| Nausea and vomiting | -0.31 | -0.22 | -0.36 | 0.34 | 1 |  |  |  |  |  |  |  |  |  |  |  |  |  |  |  |  |
| Pain | -0.41 | -0.29 | -0.43 | 0.43 | 0.29 | 1 |  |  |  |  |  |  |  |  |  |  |  |  |  |  |  |
| Dyspnea | -0.27 | -0.42 | -0.2 | 0.32 | 0.11 | 0.01 | 1 |  |  |  |  |  |  |  |  |  |  |  |  |  |  |
| Insomnia | -0.38 | -0.28 | -0.35 | 0.43 | 0.29 | 0.47 | 0.19 | 1 |  |  |  |  |  |  |  |  |  |  |  |  |  |
| Appetite loss | -0.35 | -0.39 | -0.32 | 0.57 | 0.33 | 0.29 | 0.36 | 0.27 | 1 |  |  |  |  |  |  |  |  |  |  |  |  |
| Constipation | -0.15 | -0.24 | -0.22 | 0.31 | 0.31 | 0.35 | 0.09 | 0.31 | 0.23 | 1 |  |  |  |  |  |  |  |  |  |  |  |
| BPI-3 | -0.33 | -0.2 | -0.19 | 0.26 | 0.18 | 0.7 | -0.03 | 0.37 | 0.12 | 0.26 | 1 |  |  |  |  |  |  |  |  |  |  |
| BFI-1 | -0.37 | -0.31 | -0.29 | 0.52 | 0.19 | 0.36 | 0.18 | 0.29 | 0.25 | 0.15 | 0.47 | 1 |  |  |  |  |  |  |  |  |  |
| BFI-2 | -0.4 | -0.36 | -0.33 | 0.54 | 0.17 | 0.37 | 0.18 | 0.33 | 0.29 | 0.16 | 0.45 | 0.92 | 1 |  |  |  |  |  |  |  |  |
| BFI-3 | -0.41 | -0.32 | -0.29 | 0.5 | 0.22 | 0.36 | 0.15 | 0.31 | 0.23 | 0.16 | 0.46 | 0.89 | 0.91 | 1 |  |  |  |  |  |  |  |
| BFI-4 | -0.41 | -0.45 | -0.27 | 0.51 | 0.17 | 0.35 | 0.26 | 0.32 | 0.27 | 0.25 | 0.37 | 0.7 | 0.74 | 0.75 | 1 |  |  |  |  |  |  |
| BFI-5 | -0.48 | -0.43 | -0.43 | 0.51 | 0.21 | 0.39 | 0.15 | 0.38 | 0.25 | 0.21 | 0.43 | 0.67 | 0.71 | 0.69 | 0.81 | 1 |  |  |  |  |  |
| BFI-6 | -0.41 | -0.56 | -0.26 | 0.41 | 0.1 | 0.33 | 0.29 | 0.34 | 0.2 | 0.12 | 0.41 | 0.58 | 0.64 | 0.61 | 0.67 | 0.71 | 1 |  |  |  |  |
| BFI-7 | -0.46 | -0.57 | -0.31 | 0.51 | 0.14 | 0.41 | 0.3 | 0.39 | 0.29 | 0.23 | 0.41 | 0.51 | 0.54 | 0.54 | 0.75 | 0.72 | 0.74 | 1 |  |  |  |
| BFI-8 | -0.47 | -0.45 | -0.32 | 0.45 | 0.26 | 0.33 | 0.19 | 0.4 | 0.3 | 0.21 | 0.39 | 0.5 | 0.5 | 0.48 | 0.59 | 0.69 | 0.62 | 0.69 | 1 |  |  |
| BFI-9 | -0.48 | -0.5 | -0.39 | 0.54 | 0.22 | 0.4 | 0.28 | 0.48 | 0.36 | 0.24 | 0.41 | 0.57 | 0.59 | 0.59 | 0.68 | 0.78 | 0.7 | 0.8 | 0.87 | 1 |  |
| Global fatigue score | -0.51 | -0.55 | -0.4 | 0.61 | 0.21 | 0.43 | 0.26 | 0.44 | 0.32 | 0.26 | 0.5 | 0.81 | 0.84 | 0.82 | 0.88 | 0.89 | 0.83 | 0.84 | 0.8 | 0.87 | 1 |
